# The Effect of Post-Graduate Year Training on the Self-Efficacy and Emotional Traits of Physicians Facing the COVID-19 Pandemic

**DOI:** 10.3390/healthcare9070912

**Published:** 2021-07-19

**Authors:** Chih-Hung Chen, Ya-Hui Cheng, Yuan-Chi Shen, Chia-Te Kung, Peng-Chen Chien, Ching-Hua Hsieh

**Affiliations:** 1 Department of Gastroenterology, Kaohsiung Chang Gung Memorial Hospital, Kaohsiung 833, Taiwan; totoro631105@yahoo.com.tw; 2 College of Medicine, Chang Gung University, Taoyuan City 333, Taiwan; yahui1726@cgmh.org.tw (Y.-H.C.); smallseven@cgmh.org.tw (Y.-C.S.); kungchiate@gmail.com (C.-T.K.); VENU_CHIEN@hotmail.com (P.-C.C.); 3 Graduate Institute of Adult Education, National Kaohsiung Normal University, Kaohsiung 802, Taiwan; 4 Department of Nursing, Kaohsiung Chang Gung Memorial Hospital, Kaohsiung 833, Taiwan; 5 Department of Urology, Kaohsiung Chang Gung Memorial Hospital, Kaohsiung 833, Taiwan; 6 Department of Emergency Medicine, Kaohsiung Chang Gung Memorial Hospital, Kaohsiung 833, Taiwan; 7 Department of Plastic Surgery, Kaohsiung Chang Gung Memorial Hospital, Kaohsiung 833, Taiwan

**Keywords:** severe acute respiratory syndrome, COVID-19, post-graduate year training, self-efficacy, emotional traits

## Abstract

*Background:* Taiwan implemented the post-graduate year (PGY) training to reform the medical education system to provide holistic medical care after severe acute respiratory syndrome in 2003. In late 2019, COVID-19 quickly spread across the globe and became a pandemic crisis. This study aimed to investigate whether the establishment of the PGY training had positive effects on the self-efficacy and emotional traits of medical workers. *Methods:* One hundred and ten physicians, including PGY, residents, and visiting staff, were investigated using the General Self-Efficacy Scale (GSES) and Emotional Trait and State Scale (ETSS), and their feedback and suggestions were collected. An exploratory factor analysis was done to reduce the factor dimensions using the varimax rotation method, which was reduced to four factors: “the ability to cope with ease”, “proactive ability”, “negative emotion”, and “positive emotion”. A comparison with and without PGY training when facing the COVID-19 pandemic was conducted. *Results:* Those who had received PGY training (*n* = 77) were younger, had a lower grade of seniority, and had less practical experience than those who had not received PGY (*n* = 33). Those who had received PGY training had significantly higher scores for the factors “ability to cope with ease”, “proactive ability”, and “positive emotion” than those who had not received PGY training. *Conclusion:* The study revealed that PGY training may have had positive effects on the personal self-efficacy and emotional traits of physicians coping with the COVID-19 pandemic.

## 1. Introduction

The spread of severe acute respiratory syndrome (SARS) in February 2003 raised panic and aroused high levels of alert in many medical institutions around the world. It also severely damaged Taiwan’s medical system, with some hospitals being closed, medical resources being consumed, and many employees being quarantined in the face of severe SARS nosocomial infection [1]. During the SARS epidemic, serious deficiencies in public healthcare and the medical care systems, as well as the medical education system, were also exposed. At that time, specialized physicians were lacking in training in general medical skills. The knowledge of the diagnosis, protection, and treatment for some common diseases was also ignored. Therefore, the Ministry of Health and Welfare of the Taiwan government began to implement the post-graduate year (PGY) medical training course developed by United States in 1970 [2] to reform the medical education system to strengthen resident education and the quality of medical care [3]. With the aim to provide holistic medical treatment to people, the PGY residency was designed to solve the problem of the lack of comprehensive clinical care abilities among medical graduates before their professional subdivisions [4]. In Taiwan, the three-month PGY training was implemented in 2003, progressed to six-month PGY training in 2006, and then, entered the full one-year PGY training in 2011 and two-year training in 2019 [5]. There were around 1300 PGY trainees in the training courses every year [6]. In Taiwan, participation in PGY training is necessary for every medical student to obtain a doctor’s license.

The recently emerged COVID-19 is a highly transmittable viral infection caused by a zoonotic novel coronavirus named severe acute respiratory syndrome coronavirus 2 (SARS-CoV-2) [7,8]. The disease first appeared in China in December 2019 and quickly spread across the globe [8,9]. In facing this emerging infectious disease, medical workers are not only at high risk of infection but also face a high level of psychological impact during their daily work, especially when there were still many unknowns about the spread and prevention of COVID-19 [10,11]. Many studies reported significant emotional reactions—such as depression, anxiety, and somatic symptoms—to this crisis among hospital workers [12,13]. These emotional reactions were significantly different among hospital workers from various educational backgrounds [14].

After SARS, the majority of hospital workers were increasingly aware of personal hygiene and developed a positive attitude towards dealing with emerging infectious diseases [15]. In the study of psychological impact of the COVID-19 pandemic, low perceived stress had been reported in PGY [16] and residents [17]. Therefore, it would be interesting to know whether the establishment of the PGY training has had positive effects on the self-efficacy and emotional traits of the medical workers coping with this crisis. In this study, a questionnaire regarding their feedback and suggestions was also used to obtain practical advice on the PGY training.

## 2. Materials and Methods

### 2.1. Study Design and Sample Estimation

The study included physicians aged equal to or over 20 years who worked in a hospital, a level I medical center in southern Taiwan [18,19,20]. These physicians included PGY, residents, young visiting staff (vs.), and senior vs. The young and senior vs. were defined arbitrarily by their work experience in the hospital as a vs. for less than and ≥12 years, respectively. Those who were research-related personnel or were supervisors or subordinates of the researchers were excluded from the study population. The sample size for this study was calculated using G-power statistical software. A sample size of 110 was determined by this software with setting of test family by t tests with means of difference between two independent means (two groups); the α being set to 0.05, the power to 0.70, the effect size to 0.48, and two-tailed comparison. Before proceeding with the research, this study was approved by the Institutional Review Board of Chang Gung Memorial Hospital (approval number 202000772B0). Informed consent was obtained from all patients. Before obtaining the participants’ consent and proceeding with the data collection, the researcher explained the research purpose and methods to the participants, guaranteed the confidential use of data only for research purposes, and ensured that the questionnaires would be filled in anonymously. Of 118 recruited physicians, finally, 110 (93.2%) physicians had completed the study. All methods were carried out in accordance with relevant guidelines and regulations.

### 2.2. Questionnaires for the Study 

The questionnaires for this study included four sections for the participants to fill out by themselves: (1) the personal information of the participants, (2) the General Self-Efficacy Scale (GSES), (3) the Emotional Trait and State Scale (ETSS), and (4) their feedback and suggestions (Supplemental File S1). The sociodemographic characteristics included sex, age, marital status, seniority (PGY, resident, young vs., and senior vs.), practice experience, and experience caring for patients with SARS or COVID-19. The GSES was proposed by Bandura et al. in 1997 [21] with a subsequent revised form comprising 10 questions [22]. The GSES is a single dimension and has no subscale. The participants’ feelings, thoughts, and actions were used as indicators for the assessment. The scoring was based on the Likert-type four-point scale scoring method [23] according to the suggestion for scale construction of the measures of personality and social psychological attitudes [24], with one point for completely incorrect, two points for correct, three points for mostly correct, and four points for completely correct. The total score ranged from 10 to 40 points, and a higher score indicates a better overall self-efficacy stress resistance and adjustment ability of the participant doctor. 

The ETSS originated from the Positive and Negative Affect Schedule (PANAS) to fill the need for reliable and valid measurement of positive and negative affect [25]. It consists of 28 questions regarding representative emotional traits to measure the positive and negative emotional characteristics of the participant. There are 13 questions about positive emotions and 15 about negative emotions. The positive emotional trait subscale used hope, autonomy, caring, pleasure, etc., and the negative emotional trait subscale used helplessness, inferiority complex, anger, and anxiety as the structure for the topic in the questionnaire. The scoring is based on the Likert-type four-point scale scoring method, with one point for completely incorrect, two points for correct, three points for mostly correct, and four points for completely correct. The total score ranges from 28 to 112 points. The higher the score, the better the overall positive emotional traits and state of the participant. 

The section for feedback and suggestions consisted of three open-ended questions: (1) What ability do you have to take care of patients during the COVID-19 pandemic? (2) To be competent in caring for patients with COVID-19, what kind of skills (including knowledge, attitude, and value) do you need to have besides the current training system? (3) To deal with emerging infectious diseases, what courses should be added to the PGY training?

### 2.3. Data Processing

Before starting the actual research, a pretest was first implemented with a sample of 30 individuals and presented a content validity index of 0.89 a Cronbach’s α of 0.90. The validity and internal consistency of the total questionnaire were measured with an exploratory factor analysis and Cronbach’s alpha [26]. The exploratory factor analysis was done to reduce the factor dimension via the varimax rotation method [27,28]. Kaiser–Meyer–Olkin (KMO), a test to determine sample adequacy, and Bartlett’s test of sphericity, a test to determine the degree of interrelations between variables, were used to confirm whether the data were suitable for factor analysis [29,30] under the following conditions: KMO value > 0.7, Bartlett’s test of sphericity *p*-value < 0.001, and Eigenvalue > 1. Cronbach’s alpha coefficient was used as an estimate of the internal consistency of the questionnaire. For the section on feedback and suggestions, the cumulative percentages of the answers to the questions in the section were counted and expressed as a Pareto chart of the count.

### 2.4. Statistical Analysis

The statistical analysis was performed using SPSS Windows version 23.0 (IBM Inc., Chicago, IL, USA). The categorical data were described with frequency distributions and percentages and compared using the two-sided Fisher’s exact test or Pearson’s χ2 test. The normalization of the distributed data for the continuous variables was analyzed using the Kolmogorov–Smirnov test. Analysis of variance was used with Bonferroni post hoc correction to analyze continuous data with a normal distribution. The results were expressed as mean ± standard deviation (SD). The non-normally distributed continuous data were analyzed using the Mann–Whitney *U*-test. *p* values < 0.05 indicated statistical significance.

## 3. Results

### 3.1. Basic Sociodemographic Characteristics of the Study Population

As shown in Table 1, among the 110 participants (49 female and 61 male) in this study, 77 physicians had received PGY training and 33 had not. Regarding the groups of age, seniority, and practice experience, there was a significant difference between those who had received and had not received PGY training. Because the PGY training system was set up in 2003 and most of the participants in this study aged less than 50 years, obviously, there were more participants who had received the PGY training. Further, those who had received PGY training were younger, at a lower grade of seniority, and had less practice experience than those who had not received PGY. In the same way, fewer participants who had PGY training had the experience of caring for patients with SARS than those who had not received PGY (22.1% vs. 72.7%, respectively; *p* < 0.001). In addition, fewer participants who had PGY training had the experience of caring for patients with COVID-19 than those who had not received PGY (36.4% vs. 66.7%, respectively; *p* = 0.027). 

### 3.2. Factor Analysis of the Questionnaire

Under the criteria of the loadings of the extracted factors > 0.6, the dimension of factors of the GSES and ETSS was reduced into four factors: “ability to cope with ease” (questions 1–6 of the GSES), “proactive ability” (questions 7–10 of the GSES), “negative emotion” (questions 1–14 of the ETSS), and “positive emotion” (questions 15–28 of the ETSS). The overall reliability of the scale, measured using Cronbach’s α coefficient, was 0.94. The KMO measure of sampling adequacy was 0.924 and 0.897, with Bartlett’s test of sphericity being *p* < 0.001 for the GSEG and ETSS, respectively, indicating the appropriateness of the factor analysis. 

### 3.3. Measurement of the Self-Efficacy and Emotional Traits of the Participants with Different Seniority Levels

Among the participants with different seniority levels, there was a significant difference in the factors “ability to cope with ease” and “positive emotion” with a trend that a higher level of seniority had a higher score for these factors (Table 2). Although there was no significant difference in the factors “proactive ability” and “negative emotion” among the participants with different seniority, there was a trend for the higher level of seniority to have a higher score for the factor “proactive ability” but a lower score for “negative emotion”.

### 3.4. Comparison of the Self-Efficacy and Emotional Traits of the Participants with or without PGY Training

As shown in Table 3, those who had received PGY training had significantly higher scores for the factors “ability to cope with ease”, “proactive ability”, and “positive emotion” than those who had not received PGY training. There was no significant difference regarding the factor “negative emotion” between participants who had or had not received PGY training.

### 3.5. Comparison of the Self-Efficacy and Emotional Traits of Participants with or without Experience of Caring for Patients with SARS

As shown in Table 4, those who had experienced caring for patients with SARS had significantly higher scores for the factors “ability to cope with ease”, “proactive ability”, and “positive emotion” than those who had no such experience. There was no significant difference regarding the factor “negative emotion” between participants with or without experience caring for patients with SARS.

### 3.6. Comparison of the Self-Efficacy and Emotional Traits of the Participants with or without Experience of Caring for Patients with SARS in Their Actual Caring for COVID-19 Patients

As shown in Table 5, among the 50 participants who had actually cared for COVID-19 patients, those who had experience caring for patients with SARS had significantly higher scores for the factors “ability to cope with ease”, “proactive ability”, and “positive emotion” than those who had no experience caring for patients with SARS. There was no significant difference regarding the factor “negative emotion” between participants with or without experience caring for patients with SARS.

### 3.7. Feedback and Suggestions Section

**Figure 1:**
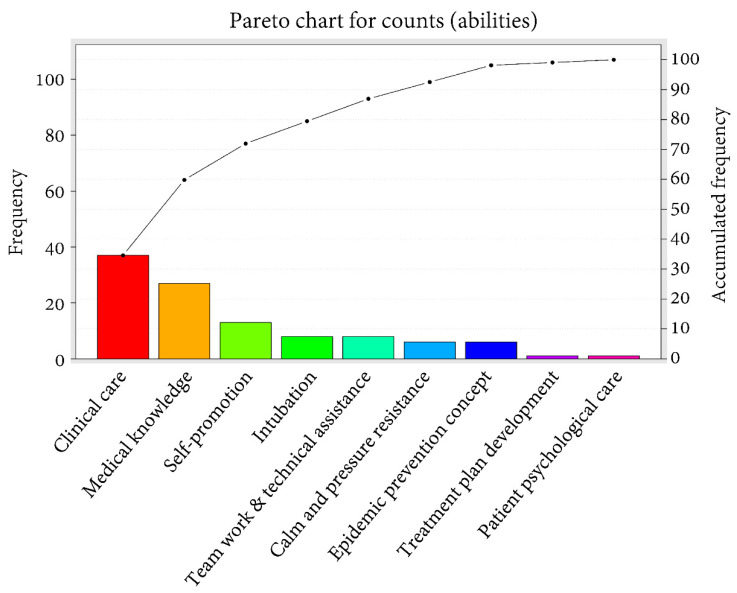
Pareto chart of the count of answers to questions regarding the abilities of the participants.

**Figure 2:**
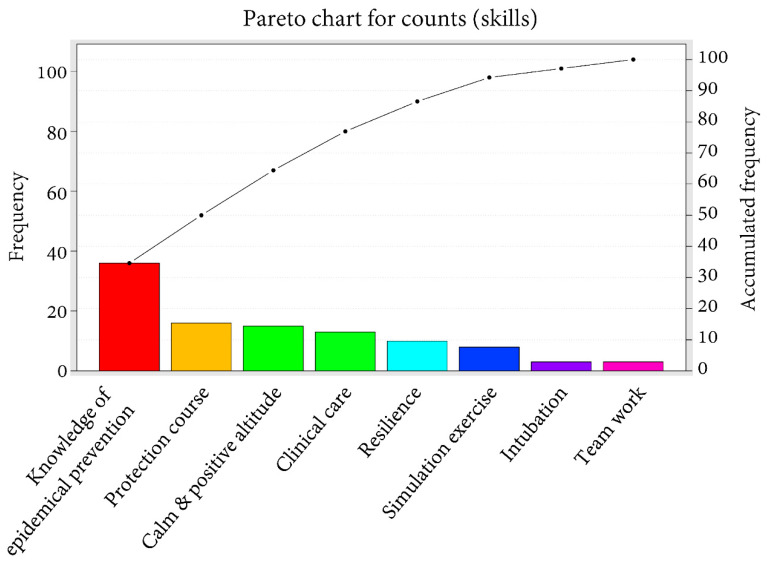
Pareto chart of the count of answers to questions regarding the skills required of the participants.

**Figure 3:**
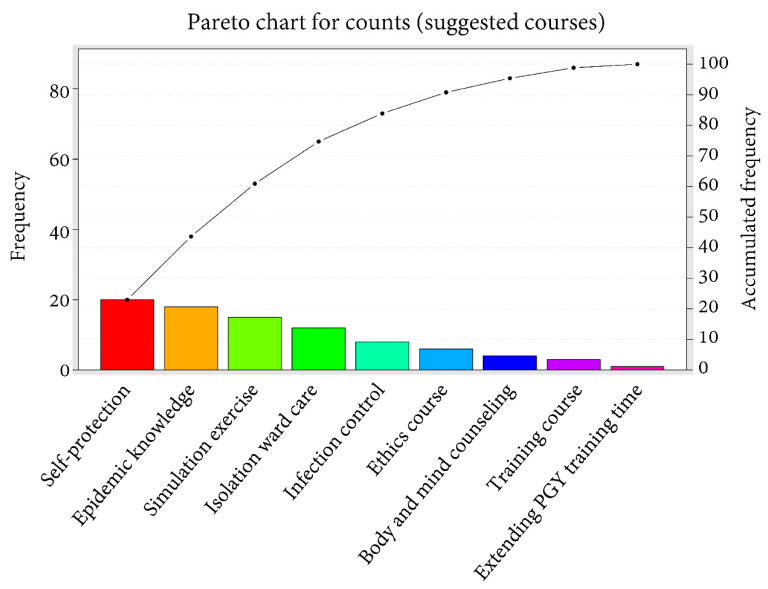
Pareto chart of the count of answers to questions regarding courses suggested to be added.

The cumulative percentages of the answers to the questions in the section for feedback and suggestions were counted and expressed as a Pareto chart of the count. Regarding the first question, “What ability do you have to take care of patients during the COVID-19 pandemic?” (Figure 1), the ability with the highest count was clinical care (*n* = 37), followed by medical knowledge (*n* = 27) with the cumulative percentage being 60%, and self-protection (*n* = 13) with the cumulative percentage being 72%. The cumulative percentage reached 99% when we included another six abilities (intubation, teamwork and technical assistance, calm and pressure resistance, epidemic prevention concept, treatment plan development, and patient psychological care). Regarding the second question on the skills required in caring for patients with COVID-19 (Figure 2), the skill with the highest count was knowledge of epidemic prevention (*n* = 37), followed by protection course (*n* = 18) with the cumulative percentage being 50%, and a calm and positive attitude (*n* = 15) with the cumulative percentage being 64%. The cumulative percentage reached 100% when we included another five abilities (clinical care, resilience, simulation exercise, intubation, and teamwork). As shown in Figure 3, to deal with emerging infectious diseases, the most recommended course for addition to the PGY training was self-protection (*n* = 25), followed by epidemic medical knowledge (*n* = 23) with the cumulative percentage being 44%, and a simulation course (*n* = 19) with the cumulative percentage being 61%. The cumulative percentage reached 100% when we included another six abilities (isolation ward care, infection control, ethics, body and mind counseling, a training course, and extending the PGY training time).

## 4. Discussion

This study demonstrated a trend where participants with a higher level of seniority had a higher score for the self-efficacy factor “ability to cope with ease” and the emotional trait factor “positive emotion”. Furthermore, although those who had received PGY training were younger, at a lower grade of seniority, and had less practical experience than those who had not received PGY, they demonstrated significantly higher scores for the factors “ability to cope with ease”, “proactive ability”, and “positive emotion” than those without PGY training, indicating the educational reform of the PGY training system has enhanced the personal self-efficacy and emotional traits of physicians facing the COVID-19 pandemic.

Many studies have shown that emotional changes brought on by the stress faced by physicians affect their physical and mental health, thereby hindering the quality of medical care and even leading to medical negligence [31,32,33]. Inappropriate emotional stress can also impact the bedside skills of health workers [31,32]. High self-efficacy can respond to and change the process of emotions during stress. In addition, personal self-efficacy also affects the expectation of results [34,35]. It has been well researched that positive emotions act as a restraining mechanism in the relationship between behavior and expected results [36,37]. People who think they have the ability to complete a task will expect a smooth and perfect result when they try to achieve a goal [35]. Furthermore, emotion is one of the sources of personal self-efficacy [38]. Positive emotions can strengthen an individual’s ability to solve problems and produce positive actions [33], even in uncertain and risky medical situations [39]. On the contrary, negative emotions suppress personal thoughts, narrow attention and cognition, and instill the belief that certain situations are difficult to control, making people afraid to act [36,37]. In this study, there was no significant difference regarding the factor “negative emotion” between participants who had or had not received PGY training. Likewise, studies in Singapore have also showed that PGY doctors [16] and residents [17] who started their clinical work reported low perceived stress in facing the COVID-19 pandemic [40]. The positive influence on the increased ability to cope with ease, emotional trait factor, and positive emotion of these trainees from this new residency system may support the value of the PGY training system on the enhancement of the personal self-efficacy and emotional traits of physicians.

The occurrence of emotional problems may be affected by multiple factors, such as a low personal tolerance threshold for pressure, inappropriate response measures, a lack of support systems, or a heavy work burden [41]. The pressure on front-line health workers in caring for SARS patients is attributed to their lack of care knowledge, experience, and protection, as well as their fear of infection [39]. Such pressure is a significant predictor of problematic health conditions [42]. This study revealed that those who had the experience of caring for patients with SARS had significantly higher scores for the factors “ability to cope with ease”, “proactive ability”, and “positive emotion” than those who had no experience in coping with the COVID-19 pandemic or in actually caring for COVID-19 patients. This information may indicate the value of redesigning core content of the PGY training to include a simulation course in caring for such patients [43]. According to the investigation of the feedback and suggestions section, a simulation course was also the third most frequently suggested course to be added to the PGY training system. Likewise, in this study, clinical care, medical knowledge, and self-protection were the top three most counted abilities that the physicians thought they possessed in caring for patients during the COVID-19 pandemic. However, epidemiological knowledge and protection are still two of the most important skills, and related courses are in high demand. This may reflect the importance of such skills in the opinions of the participants. Further, the addition of another six abilities, including isolation ward care, infection control, ethics, body and mind counseling, a training course, and extending the PGY training time, was expected to make the PGY training course more complete, implying the requirement of newer courses regarding issues of self-care, protection, and mindfulness.

## 5. Limitations of This Study

This study had some limitations. First, the results of this research based on one medical center may not be generalizable to other medical institutions. Second, in the study, not only the age, practical experience, and seniority were investigated but also the persons per se had an impact on the scores for the self-efficacy and emotional traits of the participants; however, these variables were not controlled as a matched study population to investigate the influence of PGY training on the outcome, and this may have led to some bias in the outcome measurement. Third, this study was limited by recall bias and response bias. Fourth, the content of the scale used in this study may not have covered the connotations of physician-specific self-efficacy and emotional traits. Further, with relatively small patient population, this study population was slightly underpowered as it has only 70% power. Last, the age and gender of the physicians may play a confounder for the analysis in this study. It would be better if a greater number of younger physicians without PGY training could be recruited for study. However, because most of the younger physician had received the training course since the implementation of PGY training, this goal was not able to be achieved.

## 6. Conclusions

The study revealed that PGY training may have a positive effect on the personal self-efficacy and emotional traits of physicians coping with the COVID-19 pandemic. Similar trainings could be replicated in other larger settings to have a positive impact while responding to pandemics such as COVID-19.

## Figures and Tables

**Table 1:** Sociodemographic characteristics of the study population.

Variables	PGY Training		*p*
	Yes (*n* = 77)	No (*n* = 33)	
Age (years)			<0.001
21–30, *n* (%)	52 (67.5)	3 (9.1)	
31–40, *n* (%)	18 (23.4)	6 (18.2)	
41–50, *n* (%)	7 (9.1)	16 (48.5)	
≥51, *n* (%)	0 (0.0)	8 (24.2)	
Seniority			<0.001
PGY, *n* (%)	46 (59.7)	0 (0.0)	
Resident, *n* (%)	13 (16.9)	5 (15.2)	
Junior vs., *n* (%)	15 (19.5)	6 (18.2)	
Senior vs., *n* (%)	3 (3.9)	22 (66.6)	
Practice experience			<0.001
<1 year, *n* (%)	44 (57.1)	1 (3.0)	
1–9 years, *n* (%)	24 (31.2)	7 (21.2)	
10–19 years, *n* (%)	9 (11.7)	15 (45.5)	
≥20 years, *n* (%)	0 (0.0)	10 (30.3)	
Experience caring for patients with SARS			<0.001
No, *n* (%)	60 (77.9)	9 (27.3)	
Yes, *n* (%)	17 (22.1)	24 (72.7)	
Experience caring for patients with COVID-19			0.027
No, *n* (%)	49 (63.6)	11 (33.3)	
Yes, *n* (%)	28 (36.4)	22 (66.7)	

PGY = post-graduate year; vs. = visiting staff.

**Table 2:** Measurement of the self-efficacy and emotional traits of the subjects with different seniority levels in coping with the COVID-19 pandemic. The results were expressed as mean ± standard deviation (SD).

Variables	PGY (*n* = 46)	Resident (*n* = 18)	Junior vs. (*n* = 21)	Senior vs. (*n* = 25)	*p*
Ability to cope with ease	−0.45 ± 0.97	0.07 ± 0.83	0.38 ± 0.60	0.47 ± 0.71	<0.001
Proactive ability	−0.18 ± 0.87	−0.10 ± 1.02	0.04 ± 0.75	0.37 ± 0.92	0.09
Negative emotion	0.16 ± 0.93	−0.02 ± 1.07	−0.09 ± 0.77	−0.20 ± 1.10	0.49
Positive emotion	−0.19 ± 0.99	−0.29 ± 0.89	0.23 ± 0.80	0.37 ± 0.92	0.04

**Table 3:** Measurement of the self-efficacy and emotional traits of subjects with or without PGY training in coping with the COVID-19 pandemic. The results were expressed as mean ± standard deviation (SD).

Variables	PGY Training		*p*
	Yes (*n* = 77)	No (*n* = 33)	
Ability to cope with ease	0.50 ± 0.69	−0.22 ± 0.92	0.001
Proactive ability	0.21 ± 0.86	−0.09 ± 0.81	0.009
Negative emotion	−0.07 ± 0.97	0.03 ± 0.97	0.630
Positive emotion	0.33 ± 0.92	−0.14 ± 0.94	0.020

**Table 4:** Measurement of the self-efficacy and emotional traits of subjects with or without the experience of caring for patients with SARS in coping with the COVID-19 pandemic. The results were expressed as mean ± standard deviation (SD).

Variables	Experience Caring for Patients with SARS		*p*
	Yes (*n* = 41)	No (*n* = 69)	
Ability to cope with ease	0.33 ± 0.78	−0.19 ± 0.94	0.004
Proactive ability	0.29 ± 0.79	−0.17 ± 0.92	0.006
Negative emotion	−0.17 ± 1.00	0.10 ± 0.94	0.151
Positive emotion	0.29 ± 0.90	−0.17 ± 0.95	0.014

**Table 5:** Comparison of the self-efficacy and emotional traits of subjects with or without the experience of SARS care in their actual care for COVID-19 patients.

Variables	Experience Caring for patients with SARS		*p*
	Yes (*n* = 26)	No (*n* = 24)	
Ability to cope with ease	0.29 ± 0.76	−0.16 ± 0.78	0.045
Proactive ability	0.46 ± 0.74	0.03 ± 0.58	0.026
Negative emotion	−0.23 ± 0.76	0.03 ± 0.78	0.232
Positive emotion	0.43 ± 0.87	−0.19 ± 0.72	0.009

## Data Availability

The spread sheet of de-identification data can be provided upon request only for academic research.

## Supplementary Materials

The following are available online at https://www.mdpi.com/article/10.3390/healthcare9070912/s1, Supplemental File S1: Questionnaire.
