# Supplementary material for: The Effect of Post-Graduate Year Training on the Self-Efficacy and Emotional Traits of Physicians Facing the COVID-19 Pandemic
Source: Healthcare (Basel). 2021 Jul 19;9(7):912. doi: 10.3390/healthcare9070912 (PMC8307497; doi:10.3390/healthcare9070912)
Supplement: Supplementary file 1 [file healthcare-09-00912-s001.zip › healthcare-1256452-supplementary.pdf]

Part 1. Personal Information

1. Gender: □1. Male □2. Female
2. Age (years): □1. 21–30 □2. 31–40 □3. 41–50 □4. ≥ 51
3. Marital Status: □1. Single □2. Married
4. Seniority: □1. PGY □2. Resident □3. Visiting Staff (Years______)
5. Department: □1. Family Medicine □2. Internal Medicine □3. Surgery
□4. Pediatrics □5. Obstetrics and Gynecology □6. Orthopedics
□7. Neurosurgery □8. Urology □9. Otolaryngology
□10. Ophthalmology □11. Dermatology □12. Neurology
□13. Psychiatry □14. Rehabilitation □15. Anesthesiology
□16. Radiology □17. Radiation Oncology
□18. Anatomy and Pathology □19. Clinical Pathology
□20. Nuclear Medicine □21. Emergency Medicine
□22. Occupational Medicine □23. Plastic Surgery
6. Working Experience: □1. < 1 year □2. 1–9 years □3. 10–19 years
□4. ≥ 20 years
7. SARS Care Experience: □1. Yes □2. No
8. COVID-19 Care Experience: □1. Yes □2. No

Part 2. General Self-Efficacy Scale

You faced the outbreak of the COVID-19 pandemic, where the number of confirmed cases and deaths increased sharply in Taiwan and other countries. Choose the appropriate self-assessment that describes the situation when you were taking care of COVID-19-diagnosed patients during the period. Please tick (V) the suitable box.

No.	Questions	Totally Wrong	Kind of Correct	Nearly Correct	Totally Correct
1	If I try my best, I can always solve the problem.				
2	Even if others oppose me, I can still get what I want.				
3	It is easy for me to stick to my ideals and achieve my goals.				
4	I am confident that I can deal with any sudden situations effectively.				
5	I can deal with unexpected situations				

	with my talents.				
6	Most problems will be able to be solved if I make the necessary efforts.				
7	I can face difficulties calmly because I trust my ability to deal with problems.				
8	I can usually find several solutions when facing a difficult problem.				
9	I can usually think of some ways to solve problems when in trouble.				
10	I can handle it easily no matter what happens.				

Questionnaire reference: Schwarzer (1993), revised in 1997.

Part 3. The Emotional Trait and State Scale

You faced the outbreak of the COVID-19 pandemic, where the number of confirmed cases and deaths increased sharply in Taiwan and other countries. Choose the appropriate self-assessment that describes the situation during this period. Please tick (V) the suitable box.

No.	Questions	Completely unsuitable	Nearly unsuitable	Nearly suitable	Completely suitable
1	I am often feel surprised.				
2	I am often satisfied with my own life.				
3	I often feel panic.				
4	I am often interested in people and things around me.				
5	I often feel disgusted with things around me.				

No.	Questions	Completely unsuitable	Nearly unsuitable	Nearly suitable	Completely suitable
6	I often feel calm and relaxed.				
7	I am often in a bad mood.				
8	I often feel delightful.				
9	I often feel like crying.				
10	I often think the world is beautiful.				
11	I often feel disappointed.				
12	I often feel that life is full of fun.				
13	I often feel angry.				
14	I often feel glorious.				
15	I often feel confident.				
16	I often feel guilty.				
17	I often feel lonely.				
18	I often feel that I am lucky.				
19	I often feel like others care about and trust me.				
20	I often feel happy.				
21	I often feel that my life is miserable.				
22	I often feel a lack of energy.				
23	I often feel wronged.				
24	I often feel scared.				
25	I often feel ashamed.				
26	I often feel that life is full of hope.				
27	I often feel anxious.				
28	I often feel a sense of accomplishment.				

Part 4. Feedback and Suggestions

- What ability do you have to take care of patients during the COVID-19 pandemic?

  ____

  ____

  ____

- To be competent in caring for patients with COVID-19, what kinds of skill (including knowledge, attitudes, and values) do you need to have beside the current training system?

  ____

  ____

  ____

- To deal with emerging infectious diseases, what courses should be added to the PGY training courses?

  ____

  ____
